# White matter integrity and cognitive performance in the subacute phase after ischemic stroke in young adults

**DOI:** 10.1016/j.nicl.2024.103711

**Published:** 2024-11-23

**Authors:** Mijntje M.I. Schellekens, Hao Li, Esther M. Boot, Jamie I. Verhoeven, Merel S. Ekker, Frederick J.A. Meijer, Roy P.C. Kessels, Frank-Erik de Leeuw, Anil M. Tuladhar

**Affiliations:** a Department of Neurology, Radboud University Medical Centre, Donders Institute for Brain, Cognition and Behaviour, Netherlands (the); b Department of Radiology, Radboud University Medical Centre, Donders Institute for Brain, Cognition and Behaviour, Nijmegen, Netherlands (the); c Radboud University, Donders Institute for Brain, Cognition and Behaviour, Centre for Cognition, Nijmegen, Netherlands (the); d Vincent van Gogh Institute for Psychiatry, Venray, Netherlands (the); e Department of Medical Psychology and Radboudumc Alzheimer Centre, Radboud University Medical Centre, Nijmegen, Netherlands (the)

**Keywords:** Ischemic stroke, Cognitive impairment, White matter integrity, Diffusion MRI

## Abstract

**Introduction:**

Reduced white matter integrity outside the stroke lesion may be a potential contributor of post-stroke cognitive impairment. We aimed to investigate how a stroke lesion affects the integrity of surrounding white matter, and whether the integrity of the non-lesioned part of white matter tracts is associated with cognitive performance after ischemic stroke in young adults.

**Methods:**

Patients from the ODYSSEY study, aged 18–49 years, with a first-ever ischemic stroke, underwent 3T MRI and cognitive assessment within six months after the index event. Using TractSeg and free water imaging, we analyzed free water corrected fractional anisotropy (FA_T_), free water corrected mean diffusivity (MD_T_), and free water (FW) of all white matter tracts outside the stroke lesion. We calculated FA_T_ and FW in the lesioned white matter tracts at 2 mm incremental distances from the lesion, extending up to 10 mm, represented as Z-scores using the diffusion measures of controls. We categorized patients as no/mild or major vascular cognitive disorder (VCD) and compared with a stroke-free control group (n = 23). Group differences in diffusion measures were examined. We investigated associations between FA_T_, FW and cognitive performance across seven domains.

**Results:**

Among 66 patients (median age 40.3 years (IQR 31.3–46.2); 54.5 % women), 22 had major VCD. In the different lesion expansions, we found differences in FA_T_ (p = 0.009) and FW (p = 0.049). Patients with major VCD had lower FA_T_ [range of Cohen’s d (0.65; 1.65)] and higher FW [Cohen’s d (−1.40; −0.64)] values compared to controls, both in the hemisphere affected by the lesion and the unaffected hemisphere. Performance in processing speed correlated with FA_T_ across eight tracts in the affected hemisphere [range of R^2^_adj_ (0.30; 0.37)], and with FW in four tracts in the affected and three in the unaffected hemisphere [R^2^_adj_ (0.28; 0.38)].

**Discussion:**

In the first months after a stroke, we observed a trend of microstructural changes remote from the lesion that diminish as the distance from the lesion increases. Tissue changes in the white matter outside the lesion are present in both hemispheres, but are more pronounced in the hemisphere affected by the stroke, and may contribute to worse cognitive performance.

## Highlights

• White matter changes decrease as the distance from the stroke lesion increases.
• Lower white matter integrity is more pronounced in the stroke-affected hemisphere.
• Lower white matter integrity is particularly associated with processing speed.

## 1 Introduction

Most young patients (aged 18–50 years) with an ischemic stroke experience important and lifelong consequences, including cognitive impairment as a notable outcome in almost half of these individuals (Weterings et al., 2023). Yet, the variability of cognitive impairment among young stroke patients is not completely understood. Age, education level, vascular risk factors, stroke severity, stroke location, and lesion volume are related to post-stroke cognitive impairment (Weaver et al., 2021, Pendlebury and Rothwell, 2019), but do not explain the whole range of cognitive performance and recovery after stroke at a young age (Schellekens et al., 2023). A potential contributor to post-stroke cognitive impairment may be the presence of structural changes beyond the infarcted area of the brain, referred to as remote structural changes. Previous studies from our group have shown that years after an ischemic stroke focal lesions may not only have local effects on the brain, but also extend their impact remotely throughout both hemispheres, thus impacting long-term cognitive performance (Schaapsmeerders et al., 2016). In 17 older patients with, predominantly silent, lacunar infarcts, alterations in white matter integrity have been found outside the stroke lesion. These white matter abnormalities attenuates with increasing distance to the primary lesion (Reijmer et al., 2013). In addition, studies have shown that microstructural changes in the white matter influence cognitive performance, independent of the size of the lesion (Schaapsmeerders et al., 2016, Reijmer et al., 2013, Dacosta-Aguayo et al., 2014). However, studies in the subacute phase after stroke are lacking in this young stroke population.

Given their usually long life of young stroke survivors, gaining a better understanding of the mechanisms underlying the development of poststroke cognitive impairment is important. The aim of the present study is therefore to investigate 1) how a stroke lesion affects the integrity of surrounding white matter in white matter tracts passing through the stroke lesion, and 2) whether the integrity of the non-lesioned part of the white matter tracts is associated with cognitive performance after ischemic stroke at young age.

## 2 Methods

### 2.1 Patients and study design

This study is a part of the ‘Observational Dutch Young Symptomatic Stroke Study’ (ODYSSEY) (Schellekens et al., 2023, Arntz et al., 2014). The present study included patients aged 18 to 49 years with a first-ever ischemic stroke with radiological evidence of cerebral ischemia. Patients were enrolled at the Radboudumc, Nijmegen, The Netherlands, and underwent an extensive MRI assessment, including Diffusion Weighted Imaging (DWI) protocol at baseline, along with a comprehensive neuropsychological assessment. Patients were included between December 2016 and July 2021. Exclusion criteria were a history of stroke, retinal infarction, and cerebral venous sinus thrombosis. Detailed information regarding data collection has been provided elsewhere (Arntz et al., 2014). We recruited controls among the patients’ spouses, relatives or social environment. Inclusion criteria for controls were: age between 18 and 50 years old without a history of any TIA or stroke at the moment of inclusion. We aimed for similar sex and age between controls and patients.

### 2.2 Standard protocol approvals, registrations, and patient consents

The Medical Review Ethics Committee region Arnhem-Nijmegen approved the study (NL41531.091.12). We obtained written informed consent from all participants.

### 2.3 Cognitive assessment

Patients underwent an extensive neuropsychological assessment within six months (median three months after stroke). The seven most relevant cognitive domains included: (i) Episodic memory, (ii) Processing speed, (iii) Visuoconstruction, (iv) Executive functioning, (v) Visual neglect, (vi) Language deficits, and (vii) Attention and working memory. Using normative data, raw test scores were converted to Z-scores per test for each participant, using the individual’s age, sex and/or education level. Further details regarding the collection and preparation of cognitive data can be found in the Supplementary methods section and elsewhere (Schellekens et al., 2023, Schellekens et al., 2023).

Criteria for vascular cognitive disorder (VCD) were based on the criteria of the International Society for Vascular Behavioral and Cognitive Disorders (VASCOG). We defined mild VCD as a composite Z-score of between −1.5 and −2.0 in one or more cognitive domains. We defined major VCD as a composite Z-score of <−2.0, in one more cognitive domains. Normally, mild VCD is defined as a Z-score in one or more cognitive domains between −1.0 and −2.0 (Jak et al., 2009). However, this represents 13.6 % of the normal population. By adjusting the cut-off criteria to −1.5 instead of −1.0 (representing 4.4 % of the normal population) we attain a higher specificity.

### 2.4 Other measurements

Level of education was scored with a Dutch scoring system comprising seven categories (Verhage, 1964) that align with the UNESCO international classification of education levels (UNESCO, 2011). We assessed symptoms of depression using the Mini International Neuropsychiatric Interview (MINI) (Sheehan et al., 1998) and fatigue using the subscale Subjective Fatigue of the revised Checklist Individual Strength (CIS-20R) (Vercoulen et al., 1994). We used the Barthel Index (Mahoney and Barthel, 1965) and modified Rankin Scale (mRS) (van Swieten et al., 1988) to assess functional outcome at the time of the cognitive assessment. Additionally, we evaluated the etiology of stroke (based on modified Trial of ORG 10172 in Acute Stroke Treatment; TOAST) (Hennerici and Investigators, 2009, Ekker et al., 2023) and severity at admission and discharge (National Institutes of Health Stroke Scale; NIHSS) (Brott et al., 1989), if necessary retrospectively, using a validated approach (Kasner et al., 1999, Williams et al., 2000). We visually scored markers of small vessel disease (SVD) on MRI. Evaluation of 4 markers (lacunes, microbleeds, white matter hyperintensity Fazekas score, and enlarged perivascular spaces score) led to the total SVD burden score of 0–4 (Lau et al., 2017).

### 2.5 Neuroimaging data acquisition

MRI scans were performed on a 3T MRI scanner (Siemens Magnetom Trio, Erlangen, Germany). The imaging protocol included: (i) 3D T1 magnetization-prepared rapid gradient echo (MPRAGE) (TR/TE 2300/2.3 ms; flip angle 8; voxel size 0.9 × 0.9 × 0.9 mm), (ii) 3D fluid attenuated inversion recovery (FLAIR) (TR/TE 5000/394 ms; voxel size 1.0 × 1.0 × 1.0 mm), (iii) gradient echo susceptibility weighted imaging sequence (TR/TE 27/20 ms; 3 mm slice), (iv) DWI (TR/TE 8700/67 ms; voxel size 2 × 2 × 2 mm; 3 unweighted scans, 100 diffusion-weighted scans, with non-co-linear orientation of the diffusion-weighting gradient, and b value 0.50(3×)/150(7×)/350(30×)/1000(60×) s/mm^2^).

### 2.6 Neuroimaging data processing

#### 2.6.1 Lesion segmentation

All stroke lesions were segmented semi-automatically using ITK-SNAP (Yushkevich et al., 2016). Lesions were segmented on DWI (n = 27) when identified within two weeks from the index event, on FLAIR (n = 38) if the lesion was identified after two weeks (except for one lesion which was identified after 10 days, but better visible on FLAIR), or if not available on T1 sequences (n = 1).The lesions were reviewed and manually adjusted if necessary. We calculated lesion volumes using FSLstats (Jenkinson et al., 2012). To generate a lesion probability map, all lesion masks were registered to a standard space and merged. Additional details of the normalization process can be found in the Supplementary methods section.

#### 2.6.2 Pre-processing

We pre-processed the diffusion data following these steps: (1) noise removal using the Marchenko-Pastur PCA algorithm (Veraart et al., 2016, Veraart et al., 2016, Cordero-Grande et al., 2019) and removal of Gibbs ringing artifacts (Kellner et al., 2016); (2) correction for head motion and eddy current-induced distortions (Andersson and Sotiropoulos, 2016); (3) correction for susceptibility-induced distortions using the 'topup' (using synthesized b0 image generated from the T1 image) algorithm (Andersson and Sotiropoulos, 2016); and (4) correction for B1 field inhomogeneity. After pre-processing, the first eigenvector (V1) of the diffusion map, specifically in the corpus callosum region, was visually inspected to confirm anatomical plausibility of its direction and to exclude significant artifacts. We used MRtrix 3.0 software (Tournier et al., 2019), Functional Magnetic Resonance Imaging of the Brain Software Library (FSL; v6.0.3) (Smith et al., 2004), Synb0-DISCO (Schilling et al., 2019), and Advanced Normalization Tools (ANTs, v 2.1.0) (Tustison et al., 2010) for these pre-processing steps. Due to the absence of a b0 image with reversed phase encoding in our DWI scans, ‘topup’ was performed based on a synthesized b0 image from the T1 image using Synb0-DISCO (Schilling et al., 2019).

#### 2.6.3 Free water imaging and tract specific analysis

We calculated free water corrected fractional anisotropy (FA_T_), free water corrected mean diffusivity (MD_T_), free water corrected mode of anisotropy (MO_T_), and free water (FW), using nonlinear regularised minimization process implemented in Matlab (Pasternak et al., 2009). Free water elimination might enable better estimation of FA, MD and MO by minimizing the effect of cerebrospinal fluid (CSF) and vasogenic oedema (Pasternak et al., 2009).

Next, we used TractSeg, a deep learning-based framework for automated white matter bundle segmentation, to segmentate 72 white matter fiber tracts (Wasserthal et al., 2018). For full names of the white matter tracts see Supplementary methods section. We calculated the diffusion measures in the white matter tracts outside the stroke lesion. To reduce bias, we excluded the fornix to remove unavoidable CSF partial-volume effects, as well as the striatal projections, due to a high anatomical overlap with thalamic projections (Dewenter et al., 2023). To reduce the number of comparisons, we classified tracts according to whether they were in the affected side (i.e., the side of the lesion) or unaffected side (i.e., the contralateral side) based on the lesion location. For supratentorial lesions, we regarded the infratentorial region as the unaffected side, and vice versa. For further details see Supplementary methods section. For controls, we averaged the tract measures of left and right hemispheres.

#### 2.6.4 Lesion expansion

We investigated FA_T_, MO_T_, and FW values within lesioned white matter tracts (i.e. every tract passing through the lesion) across lesion expansions of 2, 4, 6, 8, and 10 mm. The expansion distance was selected based on the voxel size of our DWI scans (2 mm), ensuring accurate value extraction for each expansion. To minimize the impact of lesion location variability, we registered the diffusion images of each control to each patient, see the Supplementary methods section. For each control (now in patient’s DWI space), we calculated the mean diffusion values at locations corresponding to the different lesion expansion distances. We calculated Z-scores for every patient for each lesion expansion distance, using the mean and standard deviation of the diffusion values of the controls. Finally, the Z-scores across all expansions within lesioned tracts were averaged for the patients, resulting in a single Z-score per expansion distance for each diffusion measure.

MRI scans were visually checked at each step of processing to ensure the accuracy of registration.

### 2.7 Statistical analysis

We compared baseline characteristics between patients with no/mild VCD versus major VCD and for patients versus controls, using the independent *t*-test or Mann-Whitney *U* test for continuous variables and Pearson’s Chi-squared/Fisher’s exact test for categorial variables. We compared Z-scores of the diffusion measures of the lesion expansions with analysis of variance (ANOVA) followed by Tukey’s HSD for post-hoc comparison. To compare differences in overall diffusion measures between controls, patients with no/mild VCD, and major VCD, we used ANOVA followed by Tukey’s HSD for post-hoc comparison. To compare differences in diffusion measures in the different white matter tracts between controls, patients with no/mild VCD, and major VCD, we used ANCOVA, controlling for depression. Missing data on depressive symptoms was imputed by the mode. Additionally, we controlled for lesion volume when comparing patients with no/mild VCD to those with major VCD. Additionally, we computed Cohen’s d as an effect size measure for group comparisons for all white matter tracts. We conducted a linear regression analysis to investigate the relationship between the diffusion measures per tract and domain-specific Z-scores, while adjusting for lesion volume and depressive symptoms. FDR correction, using the Benjamini-Hochberg procedure, was applied to p-values in all analyses involving white matter tracts, and was performed separately for each analysis involving all affected and unaffected white matter tracts. If FDR correction was applied, only results that remained FDR-significant were reported in the text.

To explore the impact of the time interval between the event and the MRI (within two weeks or thereafter) on our results, we performed post-hoc analyses for the differences in these groups for the Z-scores of the diffusion measures in the lesion expansions and for the differences in overall diffusion measures. In addition, we performed a linear regression analysis to analyze time to MRI on the overall diffusion measures. Additionally, to explore the impact of any SVD on the white matter integrity, we compared the overall diffusion measures after excluding patients with any SVD.

All statistical analyses were performed using RStudio 2022.02.01.

## 3 Results

DWI and neuropsychological assessment at baseline were available for 66 ischemic stroke patients and 23 controls. Baseline characteristics of patients and controls are described in Table 1. Median age of patients at stroke onset was 40.3 years (IQR 31.3–46.2), 54.5 % were women (n = 36). Median NIHSS at admission was 3 (IQR 1–5), median time from index event to cognitive assessment was 85 days (IQR 50–141). Imaging characteristics are presented in Table 2. Median time to MRI was 32 days (IQR 5–90) and median lesion volume was 1.00 mL (IQR 0.47–6.12). Patients with major VCD were more frequent women (p = 0.002), had a lower education level (p = 0.036), a higher NIHSS at discharge (p = 0.005), a higher mRS (p < 0.001), and a larger lesion volume (p = 0.007) compared to patients with no/mild VCD. Supplementary Fig. 1 shows an overlap of all stroke lesions.

**Table 1:** Baseline characteristics.

	Patients (n = 66)				Controls (n = 23)	p-value^b^
	Whole group (n = 66)	No/mild VCD (n = 44)	Major VCD (n = 22)	p-value^a^		
Median age, years (IQR)	40.3 (31.3–46.2)	41.3 (32.6–46.8)	38.8 (27.2–44.1)	0.495	34.5 (27.0–47.0)	0.722
Men, n (%)	30 (45.5)	26 (60.1)	4 (18.2)	**0.002**	12 (52.2)	0.578
Median time to cognitive assessment, days (IQR)	85 (50–141)	88 (48–138)	77 (55–148)	0.744	−	
Median education level (IQR)	5 (5–6)	5 (5–6)	5 (5–5)	**0.036**	6 (5–7)	**0.003**
Median NIHSS score at admission (IQR)	3 (1–5)	3 (1–5)	3 (2–4)	0.742	−	
Median NIHSS score at discharge (IQR)	0 (0–2)	0 (0–1)	2 (0–3)	**0.005**	−	
Median Barthel Index (IQR)	100 (100–100)	100 (100–100)	100 (95–100)	**0.002**	−	
Good outcome (BI ≥ 85), n (%)	60 (96.7)	41(100)	19(90.5)		−	
Median mRS (IQR)	1 (1–2)	1 (0–1)	2 (1–2)	**<0.001**	−	
Good outcome (mRS 0–1), n (%)	42 (63.6)	36 (81.8)	6 (27.2)		−	
MINI symptoms of depression present, n (%)	6 (9.2)	3 (7.0)	3 (13.6)	0.399	1 (4.3)	0.457
Mean CIS-20R − fatigue severity (SD)	33.0 (11.8)	30.7 (11.8)	37.4 (10.8)	0.059	21.5 (10.2)	**<0.001**
No/mild fatigue < 36, n (%)	26 (53.1)	19 (59.4)	7 (41.2)		20 (90.9)	
Severe fatigue ≥ 36, n (%)	23 (46.9)	13 (40.6)	10 (58.8)	0.361	2 (9.1)	**0.002**
TOAST, n (%)				0.553		
Atherothrombotic	1 (1.5)	0 (0.0)	1 (4.5)		−	
Likely atherothrombotic	3 (4.5)	2 (4.5)	1 (4.5)		−	
Small vessel disease	9 (13.6)	7 (15.9)	2 (9.1)		−	
Cardioembolic	17 (25.8)	13 (29.5)	4 (18.2)		−	
Rare causes	11 (16.7)	7 (15.9)	4 (18.2)		−	
Multiple causes	7 (10.6)	3 (6.8)	4 (18.2)		−	
Cryptogenic	18 (27.3)	12 (27.3)	6 (27.3)		−	

Education category 5, i.e. middle school/secondary vocational training. VCD: Vascular Cognitive Disorder; IQR: interquartile range; NIHSS: National Institutes of Health Stroke Scale; BI: Barthel Index; mRS: modified Rankin Scale; MINI: Mini International Neuropsychiatric Interview; CIS-20R: Checklist Individual Strength; TOAST: Trial of ORG 10172 in Acute Stroke Treatment. Missing data patients: NIHSS at discharge 2 (3.0 %); Barthel Index 4 (6.1 %); MINI symptoms of depression 1 (1.5 %); CIS-20R-fatigue 17 (25.8 %). Missing data controls: CIS-20R-fatigue 1 (4.3 %).

a p-value no/mild VCD compared to major VCD; ^b^ p-value patients compared to controls. Bold p-values represent significant differences.

**Table 2:** Imaging characteristics.

	Patients (n = 66)			
	Whole group (n = 66)	No/mild VCD (n = 44)	Major VCD (n = 22)	p-value
Median time to MRI, days (IQR)	32 (5–90)	32 (5–76)	28 (6–108)	0.522
Lesion location, n (%)				0.969
Right supratentorial	28 (42.4)	19 (43.2)	9 (40.9)	
Left supratentorial	21 (31.8)	14 (31.8)	7 (31.8)	
Bilateral supratentorial	3 (4.5)	2 (4.5)	1 (4.5)	
Infratentorial	5 (7.8)	3 (6.8)	2 (9.1)	
Unilateral supratentorial and infratentorial	2 (3.0)	2 (4.5)	0 (0.0)	
Bilateral supratentorial and infratentorial	7 (10.6)	4 (9.1)	3 (13.6)	
Median lesion volume on MRI, mL (IQR)	1.00 (0.47–6.12)	0.89 (0.38–1.90)	3.99 (0.79–14.31)	**0.007**
Median total SVD score (IQR)	0 (0–0)	0 (0–0)	0 (0–1)	0.086

VCD: Vascular Cognitive Disorder; IQR: interquartile range; SVD: small vessel disease. Bold p-values represent significant differences.

### 3.1 Lesion expansion

**Fig. 1:**
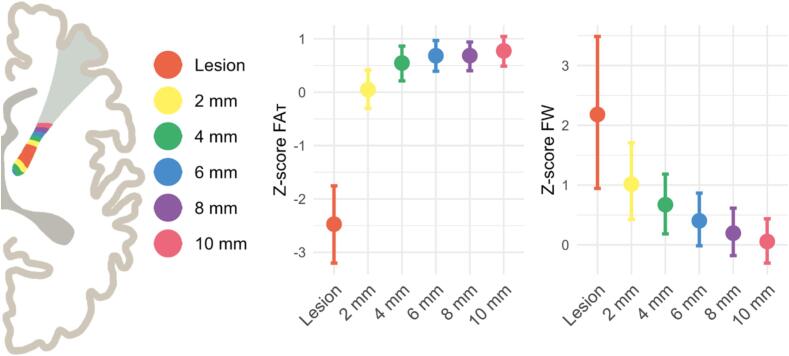
Z-scores with 95 % confidence interval of fractional anisotropy (FA_T_) and free water (FW) in lesion expansions. Schematic figure of a part of a stroke lesion within a white matter tract and the 2, 4, 6, 8, and 10 mm expansions. Z-scores for FA_T_ and FW in patients across different lesion expansions. Z-scores were calculated using the diffusion measures of controls at the same location.

Fig. 1 shows the Z-scores FA_T_ and FW for the stroke lesion and the expansions in the lesioned white matter tracts. Absolute values of FA_T_ and FW are presented in Supplementary Fig. 2. In the ANOVA models, we found differences in Z-scores of FA_T_ (p = 0.009) and FW (p = 0.049) in the different expansions. In the post-hoc analyses, the Z-scores for FA_T_ were lower in the 2 mm expansion compared with the 6 mm (p = 0.037), 8 mm (p = 0.036) and 10 mm (p = 0.011). Other post-hoc comparisons were not statistically significant. To assess whether the relatively higher FA_T_ values in patients compared to controls can be explained by the loss of crossing fibers, we examined the Z-scores of MO_T_ in the lesion expansions (Supplementary Fig. 3). We observed relatively high MO_T_ values across all lesion expansions in patients compared to controls.

### 3.2 Group comparisons of the integrity of the white matter tracts

**Fig. 2:**
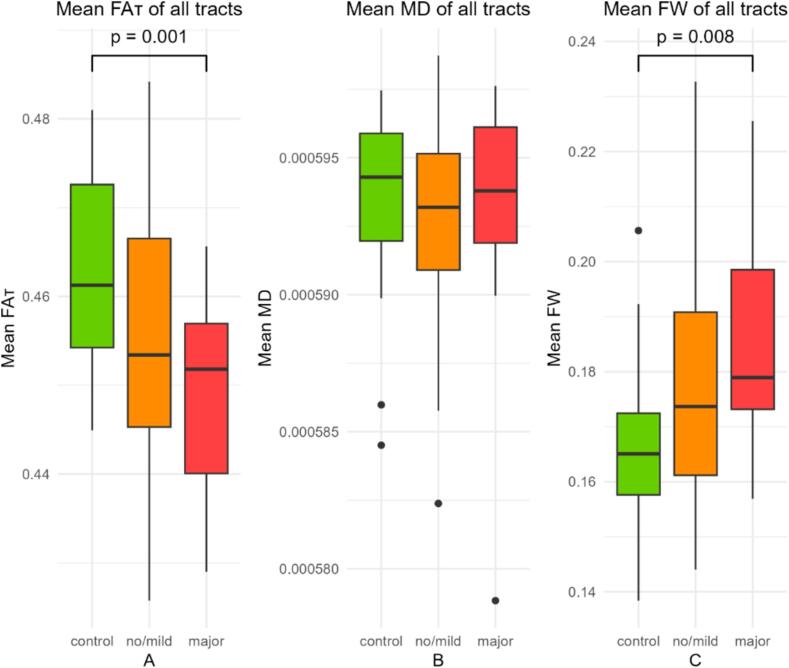
Group comparisons of overall white matter integrity. Group comparisons for controls (green), patients with no/mild VCD (orange), and major VCD (red). (A) Mean free water corrected fractional anisotropy (FA_T_) across all tracts in. (B) Mean free water corrected mean diffusivity (MD_T_) across all tracts. (C) Mean free water (FW) across all tracts.

Patients with major VCD had an overall lower mean FA_T_ (p = 0.001) and a higher mean FW (p = 0.008) compared to controls (Fig. 2A and C). For mean MD_T_, there were no significant differences between groups (Fig. 2B).

**Fig. 3:**
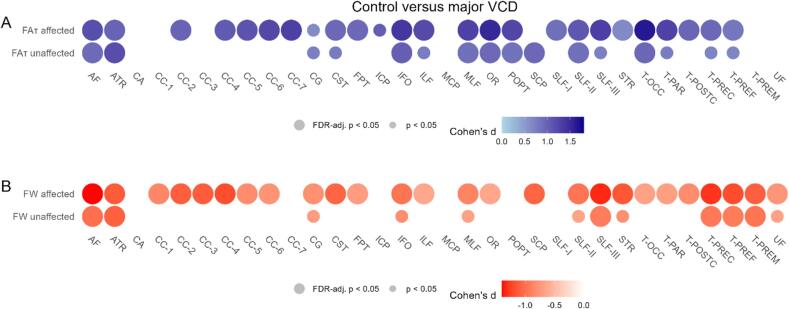
Group comparisons of free water corrected fractional anisotropy (FA_T_) and free water (FW). Difference in (A) FA_T_ and (B) FW between controls and patients with major VCD quantified with Cohen’s d represented by color. Circle size represents statistical significance level. Group differences were corrected for depressive symptoms. Group differences were presented for the tracts on the affected side, and the unaffected side. Correction for multiple comparisons was performed using false discovery rate (FDR). Large circles represent corrected p-values smaller than 0.05, small circles represent uncorrected p-values smaller than 0.05, and blank spaces represent uncorrected p-values greater than 0.05.

The major VCD group had lower FA_T_ values of most tracts on the affected side [range of Cohen’s d (0.65; 1.65), range of p-value (<0.001; 0.05)], and lower FA_T_ values of some tracts on the unaffected side than controls [Cohen’s d (0.85; 1.18), p-value (0.02; 0.03), Fig. 3A]. Similarly, the major VCD group showed higher FW values in most on the affected side [Cohen’s d (−1.40; −0.64), p-value (<0.001; 0.04)], and in some tracts on the unaffected side [Cohen’s d (−1.09; −0.92), p-values all 0.03, Fig. 3B]. The differences in FA_T_ and FW between patients with no/mild VCD versus major VCD were not significant, and the differences between controls and patients with no/mild there were only statistically significant in three tracts (all in the corpus callosum) for FW on the affected side after FDR correction (Supplementary Fig. 4).

### 3.3 Association of cognitive performance with white matter integrity

**Fig. 4:**
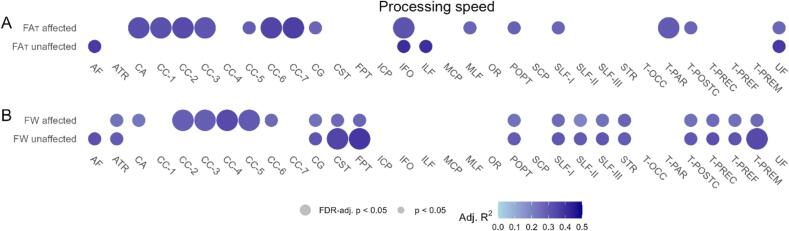
Association between processing speed and free water corrected fractional anisotropy (FA_T_), and free water (FW). Effect sizes (adj. R^2^) obtained from linear regression, adjusted for lesion volume, and depressive symptoms are presented by color. Circle size represents statistical significance level. Associations were presented for (A) the FA_T_ and (B) FW of the tracts on the affected side, and the unaffected side. Correction for multiple comparisons was performed using false discovery rate (FDR). Large circles represent corrected p-values smaller than 0.05, small circles represent uncorrected p-values smaller than 0.05, and blank spaces represent uncorrected p-values greater than 0.05.

In linear regression, processing speed was moderately associated with FA_T_ values in eight tracts on the affected side [range of R^2^_adj_ (0.30; 0.37), range of p-values (0.02; 0.05), Fig. 4A]. Processing speed was moderately associated with the FW values in four tracts on the affected side [R^2^_adj_ (0.28; 0.34), p-value 0.02;0.05)], and the FW values of three tracts on the unaffected side [R^2^_adj_ (0.33; 0.38), p-value (0.01; 0.04), Fig. 4B]. Associations between the other cognitive domains, except for language deficits, and FA_T_ and FW were observed in various tracts. However, the associations, except for two tracts in executive functioning, were not statistically significant after FDR correction (Supplementary Fig. 5).

### 3.4 Post-hoc analyses

We performed post-hoc analyses to investigate whether the timing of MRI was related to the diffusion parameters. We divided the group based on the timing of MRI: within 14 days after the stroke (n = 28) or thereafter. These analyses showed no significant differences in the Z-scores of FA_T_ and FW of lesion expansions, or the comparisons of the overall tract measures. In linear regression, time to MRI was not significantly associated with the overall diffusion measures.

Excluding patients with any SVD (n = 15) on their MRI did not influence the results of the group comparisons of overall white matter integrity.

## 4 Discussion

In this study of patients with a young ischemic stroke, we observed a trend that FA_T_ gradually increases, while FW gradually decreases along the lesioned white matter tracts, extending away from the stroke lesion. Furthermore, we found that lower white matter integrity outside the stroke lesion, which was more pronounced in the hemisphere affected by the stroke, is associated with worse cognitive performance, particularly in the cognitive domain processing speed.

In the present study, we observed diffusivity changes in white matter tracts remote from the stroke lesion that were not visible on conventional MRI. FA_T_ gradually increases with increasing distance from the lesion. However, only the FA_T_ in de 2 mm expansion was significantly lower than in the other expansions. The fact that this first rim around the lesion had lower FA_T_ values could be a partial volume effect. Interestingly, FA_T_ values in the expansions of the patients were higher compared to those in the same locations in the controls. This may be due to the degeneration of one or more fiber bundles in crossing fibers, resulting in a paradoxical increase in FA_T_ compared to controls (Figley et al., 2021). The combination of higher values of FA_T_ and higher values of MO_T_ in the lesion expansions in our patients supports the notion that the increase in FA_T_ is most likely explained by the selective loss of fibers in the crossing-fiber areas. The extent of increased FW diminishes as the distance from the stroke lesion increases. However, in post-hoc analyses there were no differences between the different expansions. Our findings are consistent with a previous stroke study conducted in 17 older participants, predominantly with silent infarcts (Reijmer et al., 2013). Although the confidence intervals of the Z-scores for FA_T_ and FW for most expansions overlap, our findings suggest that the process of microstructural changes might already begin in the first months after stroke. In our post-hoc analysis, we found no difference in white matter integrity between patients with a MRI scan within 14 days versus those scanned thereafter (up to six months). Additionally, the time to MRI was not associated with white matter integrity. Since all the MRI scans were made relatively early in the course after the event, a longer interval between the event and the MRI might reveal more pronounced differences in FA_T_ in the various lesion expansions.

In group comparisons, patients with major VCD exhibited lower FA_T_ values and higher FW compared to controls. When examining specific white matter tracts, we observed that this finding was not only present in the hemisphere affected by the stroke, but even in the unaffected hemisphere. While other group comparisons did not reach statistical significance, it appears that controls have the highest white matter integrity, followed by patients with no or mild VCD, and finally, those with major VCD. While direct data linking FW to physiological changes are lacking, prior research has suggested that FW changes may be an early marker of white matter damage. Although the exact mechanisms underlying FW increases are unclear, several studies suggested that the FW changes reflect pathologic processes, including neuroinflammatory processes, blood–brain barrier dysfunction, vasogenic edema and intramyelinic vacuolization (Pasternak et al., 2015, Pasternak et al., 2012).

The pattern of reduced FA_T_ outside the stroke lesion is likely also not pathophysiology specific, but may be due to potential mechanisms, such as axonal degeneration and demyelination (Reijmer et al., 2013, Kancheva et al., 2022). Secondary degeneration occurs near the lesion side and spreads along the entire length of a damaged tract (Kancheva et al., 2022), potentially extending to remote tracts. Prior stroke studies demonstrated reduced white matter integrity compared with controls in both ipsi- and contralesionally in the early phase after stroke (Egorova et al., 2020) and even years after stroke (Egorova-Brumley et al., 2023).

In contrast to the relatively high FA_T_ values in the lesion expansions, we observed an overall lower FA_T_ in patients. Another possible explanation is that the differences between groups, with respect to white matter integrity, were already present before the occurrence of the stroke. Individuals with greater cognitive reserve may have more robust white matter integrity (Stern, 2002). This may relate to the significant difference in education level in our study population, as white matter integrity is positively correlated with intelligence, for which educational attainment is a proxy (Deary et al., 2010). Additionally, lower intelligence might be associated in behaviors across the life-course that have impact on the white matter integrity (Deary et al., 2010, Gow et al., 2012). These behaviors may, for example, lead to lower white matter integrity in the normal appearing white matter.

Another explanation for the lower white matter integrity beyond the stroke lesion could be the presence of SVD that is not visible on conventional structural MRI (Ter Telgte et al., 2018). Previous studies have suggested that focal SVD lesions represent only a fraction of the underlying pathology (Maniega et al., 2015). Since visible markers of SVD were not sufficiently explanatory for the differences in white matter integrity between groups in our study, it could be that these do not fully capture the burden of SVD-related brain damage.

Moreover, we investigated the association of white matter integrity with cognitive performance in specific domains. We found an association of FA_T_ and FW with processing speed performance in tracts in the affected hemisphere and for FW even in some tracts in the unaffected hemisphere. Findings were independent of the lesion volume and depressive symptoms. An earlier study in young stroke patients also found an association between FA and processing speed, even in the unaffected hemisphere (Schaapsmeerders et al., 2016). This may be explained by the fact that this study was performed almost 11 years after ischemic stroke. Since our scans were made approximately one month after the stroke, it is possible that secondary neurodegeneration may be ongoing at this point in time. Another study in older stroke patients also reported that white matter degeneration was associated with worse cognitive performance in multidomain impairments (Egorova-Brumley et al., 2023). However, the question remains whether changes in white matter structure are directly causing cognitive impairment, whether they are an epiphenomenon, or possibly could even reflect a decrease in brain efficiency. In our study, we demonstrated that processing speed cannot be attributed to a specific tract or region, in agreement with the notion that it is a distributed cognitive ability that depends on the integrity of many anatomically widespread white matter tracts (Penke et al., 2010). We only found associations between white matter integrity and processing speed and not for other cognitive domains. It is known that processing speed is a sensitive marker of white matter damage (Bucur et al., 2008).

Our study has several notable strengths. First, the prospective study design results in reliable information. While causal relationships cannot be established, these findings offer valuable insights into the underlying cerebral mechanisms. Second, the inclusion of patients with radiologically confirmed ischemic stroke ensured the absence of misdiagnosed patients. Third, we used extensive neurocognitive testing. Fourth, we used TractSeg for tract segmentation, which has a high accuracy in segmenting white matter tracts, thus enhancing the reliability of our study’s findings. Finally, we used free water imaging, which might enable better estimation of diffusion measures by minimizing the effect of CSF and vasogenic oedema.

However, there are several limitations that need to be addressed. First, the inclusion of patients from a tertiary care setting may have introduced a potential form of selection bias. Second, stroke severity, as measured by NIHSS and mRS, was relatively mild in our cohort. Therefore, our findings may not fully represent the complete range of the young stroke population. Third, we lacked information on pre-stroke cognitive functioning. Nonetheless, considering the relatively young age of our participants and the limited presence of other comorbidities, such as pre-existing vascular or degenerative disorder in this age group, the impact of pre-stroke cognitive functioning on post-stroke cognitive functioning is likely to be minimal. Fourth, MRI scans were not conducted at exactly the same time after the ischemic event. This variability in timing may have potentially resulted in differences in microstructural changes at each timepoint. Fifth, the study may have insufficient power to detect statistically significant results. This could be attributed to either the relatively small sample size, the limited variability in diffusion metrics and the timing of the scanning. Lastly, our study is based on baseline neuroimaging data. While these provide valuable insights, longitudinal imaging data could offer a more comprehensive understanding of cerebral mechanisms and their relation with cognitive performance over time. Future research should prioritize the utilization of longitudinal imaging data, including more participants, and try to develop a prediction model for identifying patients at risk of post-stroke cognitive impairment.

In conclusion, among young stroke survivors, tissue changes in the white matter, especially in the affected hemisphere, are already present and may contribute to cognitive impairment in the subacute phase after stroke. These findings could provide valuable insights for identifying individuals at risk of cognitive impairment after a stroke and contribute to more effective risk stratification. Ultimately, this could facilitate early cognitive therapy, potentially improving outcomes by addressing cognitive impairments sooner in the rehabilitation process. In the future, it may also enable more targeted cognitive interventions during rehabilitation.

## Declaration of Generative AI and AI-assisted technologies in the writing process

During the preparation of this work the author(s) used ChatGPT in order to improve the language of the manuscript. After using this tool, the author(s) reviewed and edited the content as needed and take(s) full responsibility for the content of the published article.

## CRediT authorship contribution statement

**Mijntje M.I. Schellekens:** Writing – original draft, Visualization, Methodology, Formal analysis. **Hao Li:** Writing – review & editing, Methodology, Formal analysis. **Esther M. Boot:** Writing – review & editing. **Jamie I. Verhoeven:** Writing – review & editing. **Merel S. Ekker:** Writing – review & editing. **Frederick J.A. Meijer:** Writing – review & editing. **Roy P.C. Kessels:** Writing – review & editing. **Frank-Erik de Leeuw:** Writing – review & editing, Supervision, Funding acquisition. **Anil M. Tuladhar:** Writing – review & editing, Supervision, Project administration, Methodology, Funding acquisition, Formal analysis, Conceptualization.

## Funding

This research received no specific grant from any funding agency in the public, commercial, or not-for-profit sectors.

## Declaration of competing interest

The authors declare the following financial interests/personal relationships which may be considered as potential competing interests: A.T. is a junior staff member of the Dutch Heart Foundation (grant number 2016T044); F.E.d.L. is a Clinical established investigator of the Dutch Heart Foundation (2014T060).

## Data Availability

Anonymized data not published within this article will be made available by request from any qualified investigator after permission of regulatory bodies and medical ethics committees.
