# Supplementary material for: White matter integrity and cognitive performance in the subacute phase after ischemic stroke in young adults
Source: Neuroimage Clin. 2024 Nov 23;45:103711. doi: 10.1016/j.nicl.2024.103711 (PMC11647214; doi:10.1016/j.nicl.2024.103711)
Supplement: Supplementary Data 1 [file mmc1.docx]

**Supplementary Methods**

**Cognitive assessment**The seven cognitive domains were assessed using multiple tests: *Episodic memory* (3-trial version of the Rey Auditory Verbal Learning Test), *Processing speed* (the written version of the Symbol-Digit Modalities Test, the abbreviated Stroop Color Word Test, parts I and II), *Visuoconstruction* (Rey-Osterrieth Complex Figure (ROCF)-copy trial), *Executive functioning* (Fluency test, Stroop interference score, Brixton Spatial Anticipation Test), *Visual neglect* (Star Cancellation of the Behavioral Inattention Test), *Language deficits* (Short Token Test), *Attention and working memory* (Digit Span subtest from the Wechsler adult Intelligence Scale – Fourth Edition). Normative data from the Advanced Neuropsychological Diagnostics Infrastructure (ANDI), which includes data of 26,000 healthy individuals across all age groups were employed for most tests. This allowed fine-grained adjustment based on age, sex and education level. For the written version of the Symbol-Digit Modalities Test (Smit A, 2010), we used the normative data from the test’s manual. We used healthy controls from another stroke study for the Star Cancellation test (Nys GM et al., 2006).

**Normalization**We first registered brain-extracted FLAIR images and DWI, along with the lesion mask, to brain-extracted T1-weighted images using the Functional MRI of the Brain Linear Image Registration Tool (FLIRT). Next, these T1-weighted images were registered, along with the lesion mask, to the Montreal Neurological Institute (MNI) standard space 152 template using FLIRT, followed by nonlinear registration using the Functional MRI of the Brain nonlinear registration tool (FNIRT). We used FSL 6.0.5 tools (Jenkinson M et al., 2012).

**White matter tracts**Anterior thalamic radiation (ATR), Arcuate fascicle (AF), Commissure anterior (CA), Corpus callosum (Rostrum (CC-1), Genu (CC-2), Rostral body (CC-3), Anterior midbody (CC-4), Posterior midbody (CC-5), Isthmus (CC-6), Splenium (CC-7)), Cingulum (CG), Corticospinal tract (CST), Fronto-pontine tract (FPT), Inferior cerebellar peduncle (ICP), Inferior occipito-frontal fascicle (IFO), Inferior longitudinal fascicle (ILF), Middle cerebellar peduncle (MCP), Middle longitudinal fascicle (MLF), Optic radiation (OR), Parieto-occipital pontine (POPT), Superior cerebellar peduncle (SCP), Superior longitudinal fascicle I (SLF-I), Superior longitudinal fascicle II (SLF-II), Superior longitudinal fascicle III (SLF-III), Superior thalamic radiation (STR), Uncinate fascicle (UF), Thalamo-occipital (T-OCC), Thalamo-parietal (T-PAR), Thalamo-postcentral (T-POSTC), Thalamo-precentral (T-PREC), Thalamo-prefrontal (T-PREF), Thalamo-premotor (T-PREM), Uncinate fascicle (UF).

**Registration of DWI of controls to DWI of patients**We first registered DWI of controls to their brain-extracted T1-weighted imaged using the FLIRT. Next, brain-extracted T1-weighted images of controls were registered to the brain-extracted T1-weighted images of patients, along with the lesion mask of patients, using FLIRT, followed by FNIRT. Subsequently, spatial transformations were applied to convert DWI data from controls in T1 space of controls to the T1 space of patients. To enable the transformation of T1 space in patients to DWI space in patients, we first computed the inverse transformation matrix. Finally, we used FLIRT for the transformation of DWI of controls in T1 space of patients to the DWI space of patients. We used FSL 6.0.5 tools (Jenkinson M et al., 2012).

**Dividing tracts ass in the affected or unaffected side based on the lesion location**

Patients with supratentorial lesions:

- For unilateral supratentorial lesions, the supratentorial tracts at the side of the lesion are considered as affected, and the tracts at the contralateral side are considered as unaffected.
- For bilateral supratentorial lesions, the average of tract measures of the left and right hemisphere are considered affected, and there are no unaffected supratentorial tracts.
- All infratentorial tracts are considered unaffected, in case of two-sided tracts, the tract measures of left and right were averaged.
- Corpus Callosum I-VII and anterior commissure were considered as affected.

Patients with infratentorial lesions:

- All supratentorial tracts are considered unaffected, in case of two-sided tracts, the tract measures of left and right were averaged.
- All infratentorial tracts are considered affected, in case of two-sided tracts, the tract measures of left and right were averaged.

Patients with supra- and infratentorial lesions:

- For unilateral supratentorial lesions, the supratentorial tracts at the side of the lesion are considered as affected, and the tracts at the contralateral side are considered as unaffected.
- For bilateral supratentorial lesions, the average of tract measures of the left and right hemisphere are considered affected, and there are no unaffected supratentorial tracts.
- All infratentorial tracts are considered affected, in case of two-sided tracts, the tract measures of left and right were averaged.
- Corpus Callosum I-VII and anterior commissure were considered as affected.

Controls:

- In case of two-sided tracts, the tract measures of left and right were averaged.
