# Supplementary material for: White matter integrity and cognitive performance in the subacute phase after ischemic stroke in young adults
Source: Neuroimage Clin. 2024 Nov 23;45:103711. doi: 10.1016/j.nicl.2024.103711 (PMC11647214; doi:10.1016/j.nicl.2024.103711)
Supplement: Supplementary Data 2 [file mmc2.docx]

**Supplementary Figure 1**

**
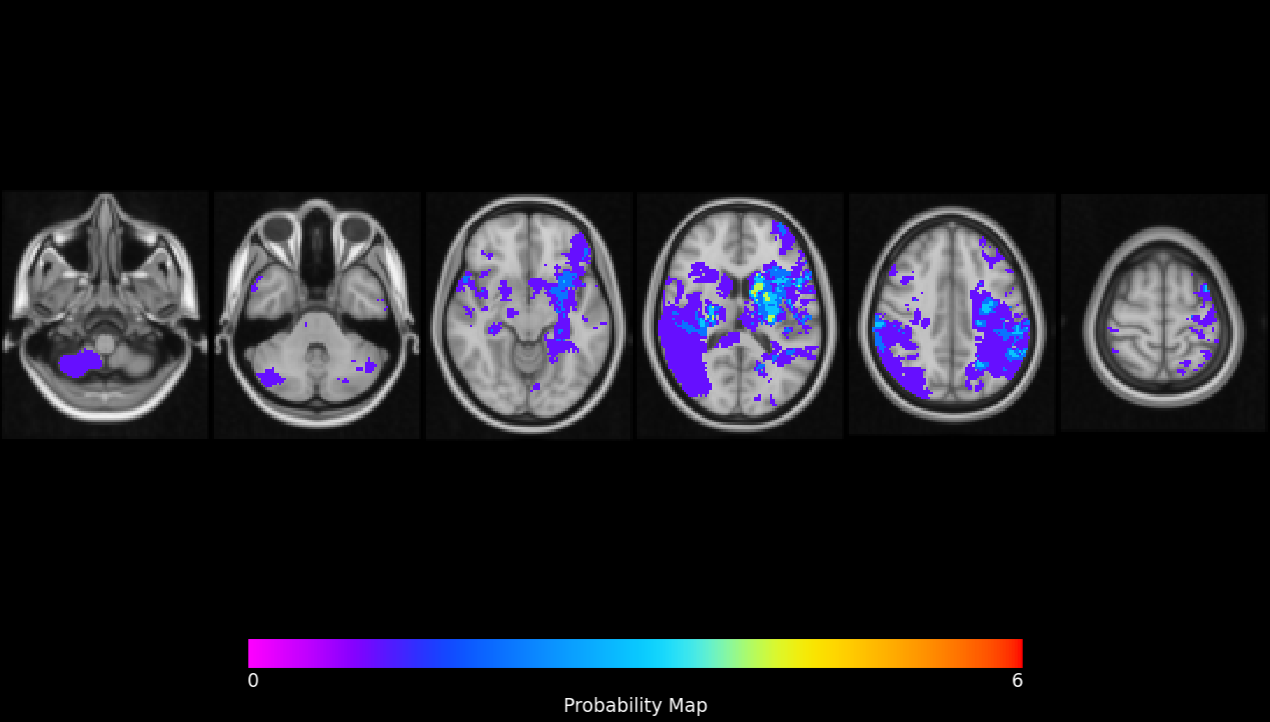
**

**Overlap of all stroke lesions in radiological orientation.** This map displays an overlap across all stroke lesion by 6 slices at z=-60, z=-35, z =-10, z=15, z=40, and z=65. The color bar is ranging from 0 to 6, indicating the number of patients with a stroke lesion in that region
