# Supplementary material for: White matter integrity and cognitive performance in the subacute phase after ischemic stroke in young adults
Source: Neuroimage Clin. 2024 Nov 23;45:103711. doi: 10.1016/j.nicl.2024.103711 (PMC11647214; doi:10.1016/j.nicl.2024.103711)
Supplement: Supplementary Data 3 [file mmc3.docx]

**Supplementary Figure 2**

***
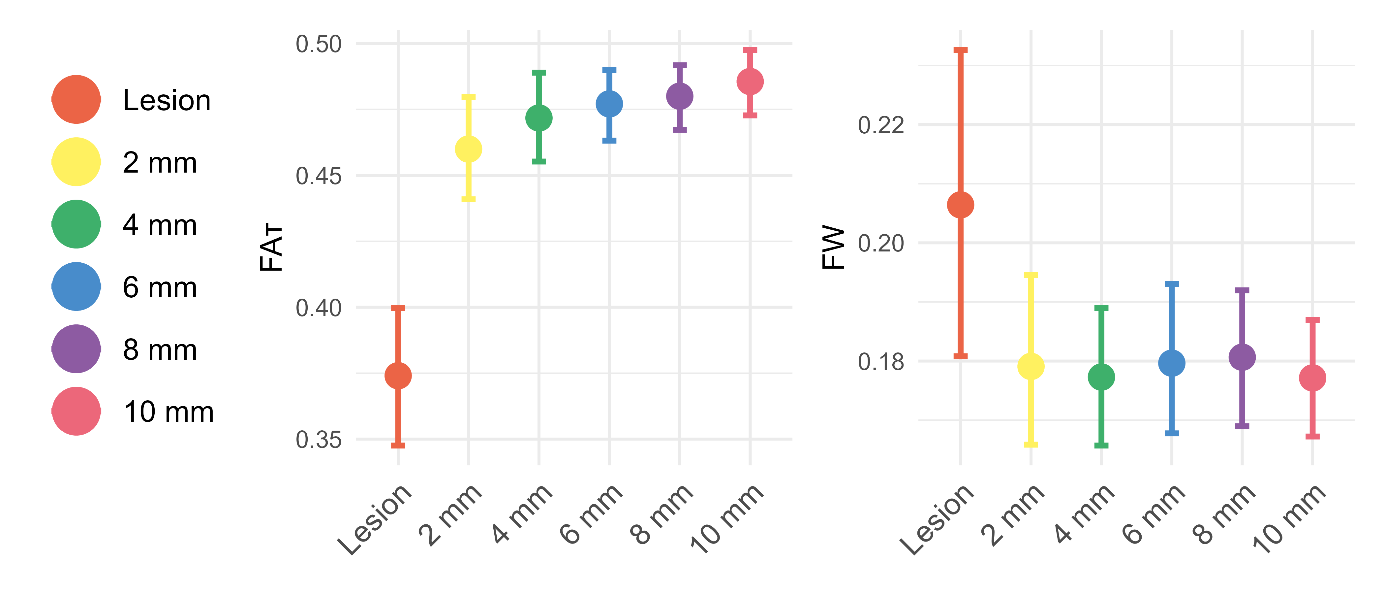
***

**Absolute values of free water corrected Fractional Anisotropy (FA_T_) and Free Water (FW) (with 95% confidence interval) in lesion expansions of patients.**
