# Supplementary material for: White matter integrity and cognitive performance in the subacute phase after ischemic stroke in young adults
Source: Neuroimage Clin. 2024 Nov 23;45:103711. doi: 10.1016/j.nicl.2024.103711 (PMC11647214; doi:10.1016/j.nicl.2024.103711)
Supplement: Supplementary Data 4 [file mmc4.docx]

**Supplementary Figure 3**


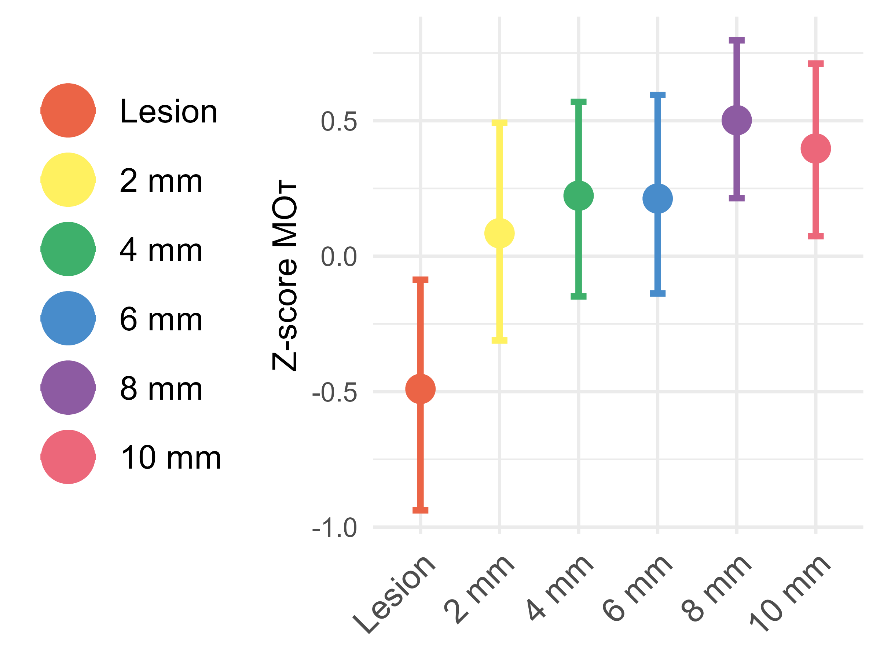


**Z-score with 95% confidence interval of free water corrected Mode of anisotropy (MO_T_) in lesion expansions.** Z-scores for MO_T_ in patients across different lesion expansions**.** Z-scores were calculated using the MO_T_ of controls at the same location).
