# Supplementary material for: White matter integrity and cognitive performance in the subacute phase after ischemic stroke in young adults
Source: Neuroimage Clin. 2024 Nov 23;45:103711. doi: 10.1016/j.nicl.2024.103711 (PMC11647214; doi:10.1016/j.nicl.2024.103711)
Supplement: Supplementary Data 5 [file mmc5.docx]

**Supplementary Figure 4**

*
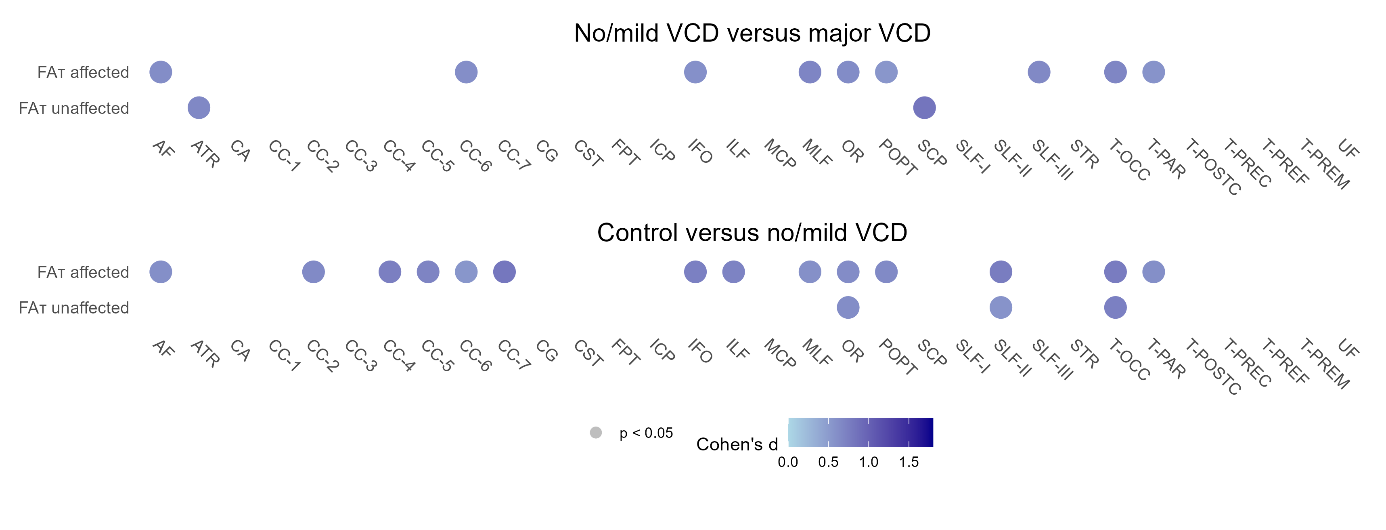
*

*
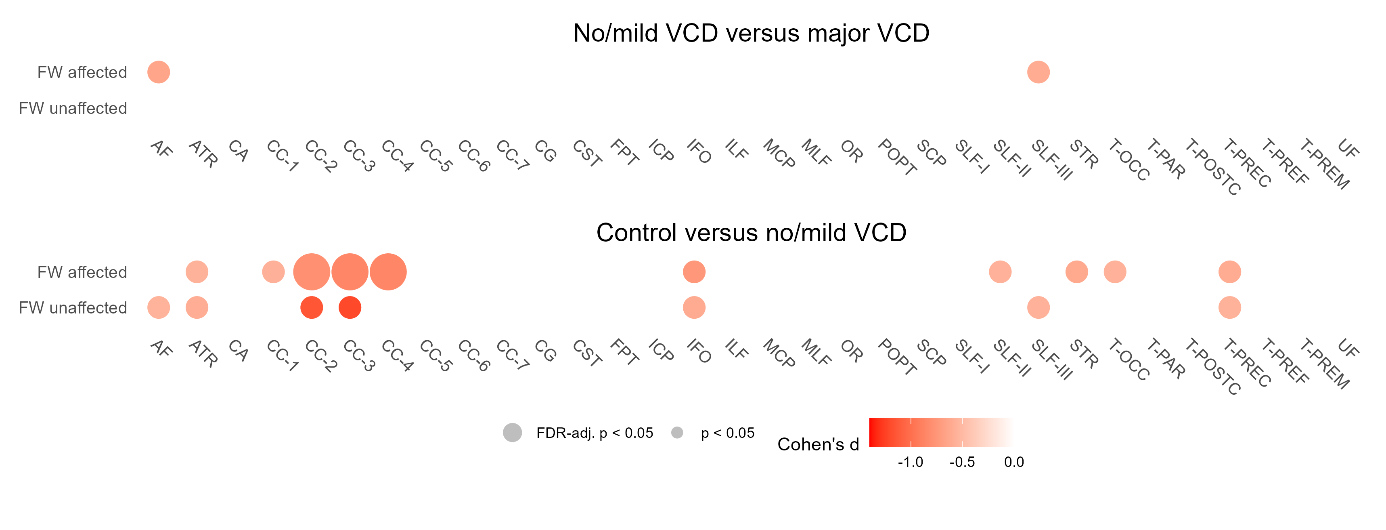
*

**Group comparisons of free water corrected Fractional Anisotropy (FA_T_) (blue) and Free Water (FW) (red).** Difference in FA_T_ and FW between patients with no/mild VCD and major VCD, and between controls and patients with no/mild VCD and quantified with Cohen’s d represented by color. Group differences were corrected for depressive symptoms and lesion volume. Group differences were presented for the FAT and FW of the tracts on the affected side, and the FAT and FW of the tracts on the unaffected side. Correction for multiple comparisons was performed using false discovery rate (FDR). In this figure circles are uncorrected p-values smaller than 0.05, and blank spaces represent uncorrected p-values greater than 0.05.
