# Supplementary material for: White matter integrity and cognitive performance in the subacute phase after ischemic stroke in young adults
Source: Neuroimage Clin. 2024 Nov 23;45:103711. doi: 10.1016/j.nicl.2024.103711 (PMC11647214; doi:10.1016/j.nicl.2024.103711)
Supplement: Supplementary Data 6 [file mmc6.docx]

**Supplementary Figure 5**

*
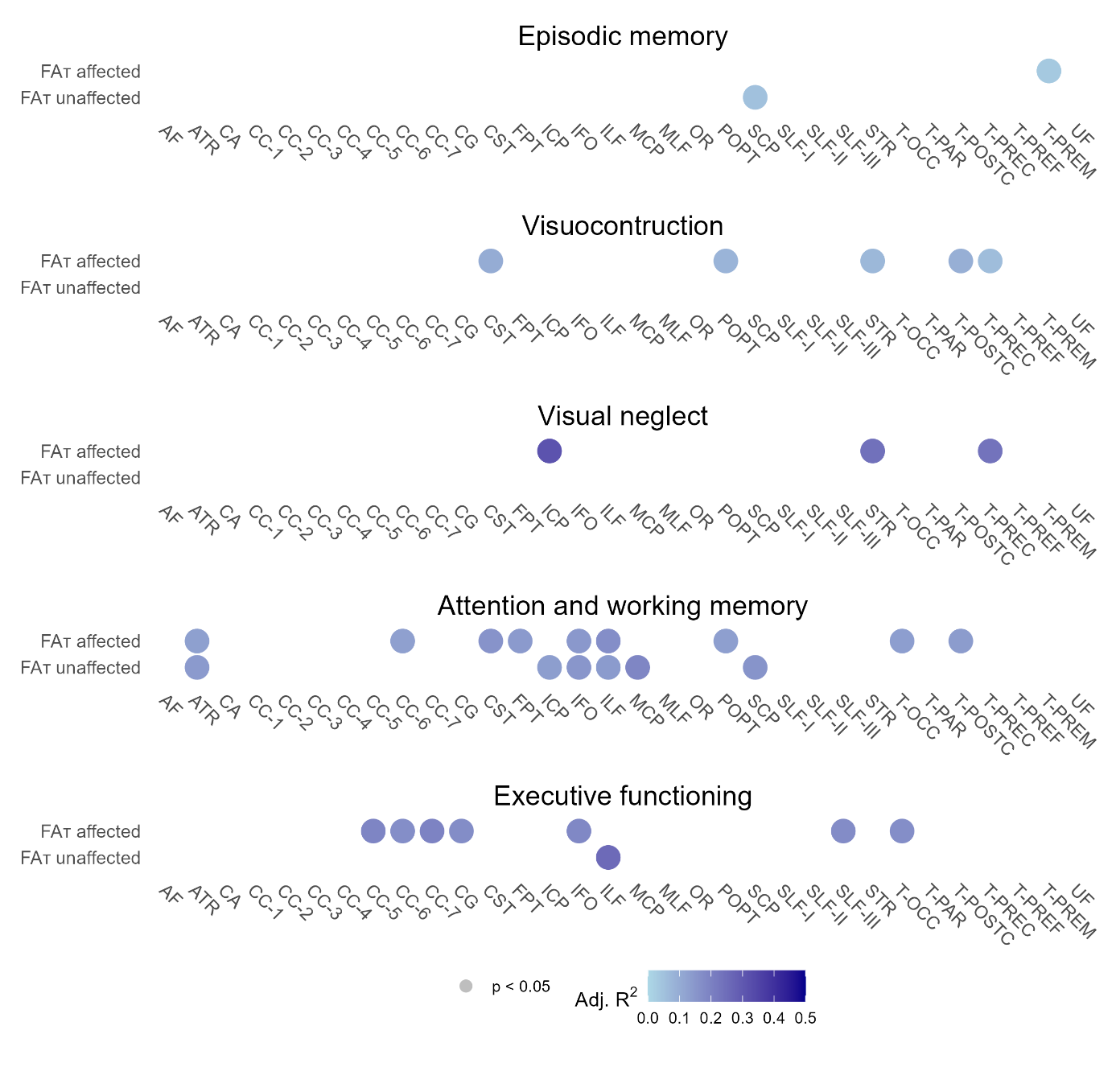
*

***
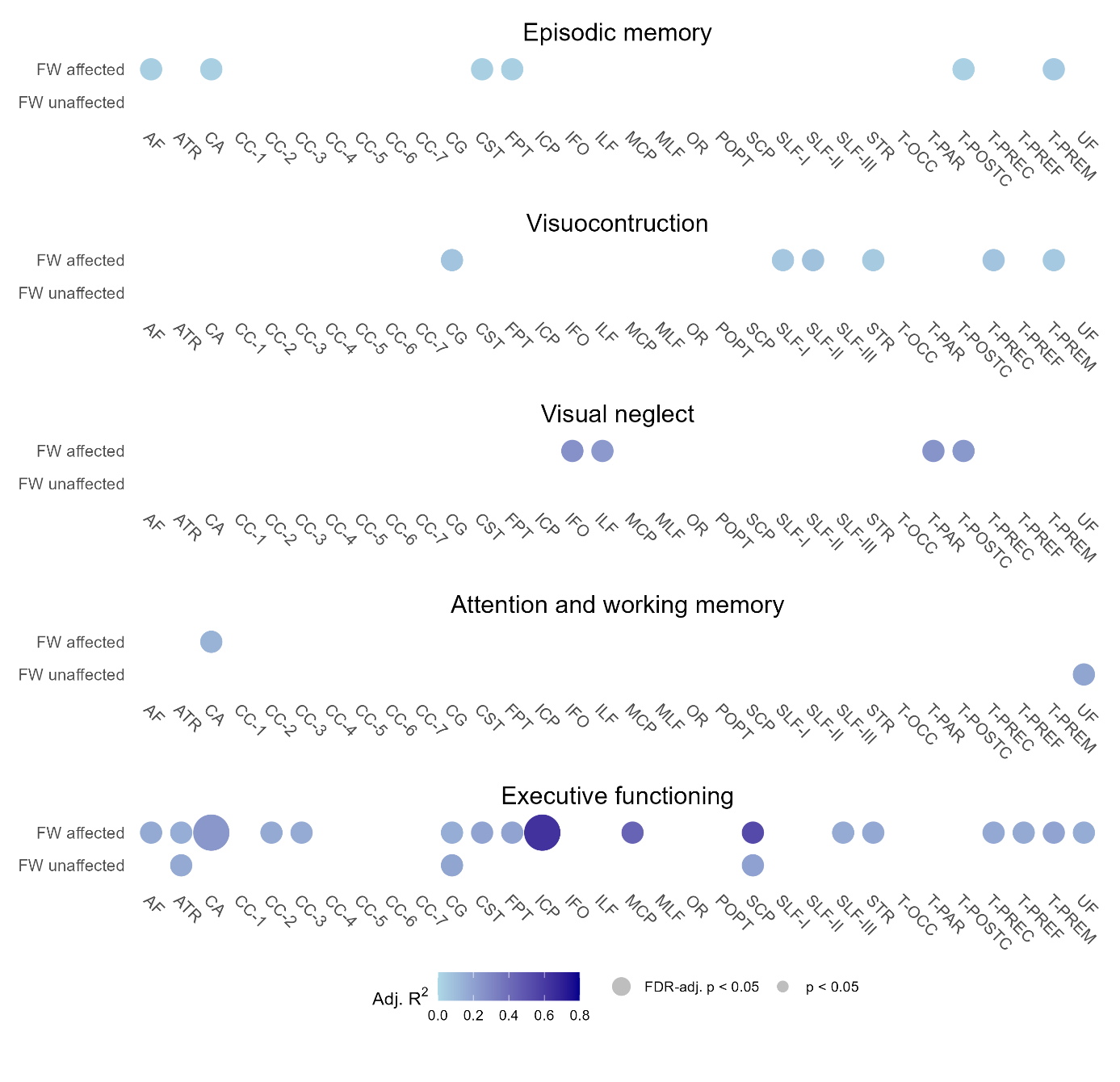
*Association between domain specific z-scores and free water corrected Fractional Anisotropy (FA_T_), and Free Water (FW).** Effect sizes (adj. R^2^) obtained from linear regression, adjusted for lesion volume and the presence of depressive symptoms are presented by color. Associations were presented for the FA_T_ and FW of the tracts on the affected side, and the FA_T_ and FW of the tracts on the unaffected side. Correction for multiple comparisons was performed using false discovery rate (FDR). Large circles represent corrected p-values smaller than 0.05, small circles represent uncorrected p-values smaller than 0.05, and blank spaces represent uncorrected p-values greater than 0.05
